# Health status of the population in Naqu, Tibet and its latent class analysis: a cross-sectional survey

**DOI:** 10.3389/fpubh.2023.1223382

**Published:** 2023-11-03

**Authors:** Jiaxue Cui, Ouzhu Nima, Duoji Zhaxi, Chenxin Jin, Ruiqi Wang, Yizhuo Diao, Yongxing Chen, Xiaoguang Xu, Xiaofeng Li

**Affiliations:** ^1^Department of Epidemiology and Health Statistics, Dalian Medical University, Dalian, China; ^2^Institute of High Altitude Medicine, People’s Hospital of Naqu Affiliated to Dalian Medical University, Naqu, China; ^3^Department of Neurosurgery, Second Affiliated Hospital of Dalian Medical University, Dalian, China

**Keywords:** Tibet, population health, latent class analysis, cross-sectional survey, herders and farmers, health knowledge

## Abstract

**Background:**

Through a survey and analysis of the population’s present state of health, it is possible to give data support for improving the health status of inhabitants in Naqu, Tibet. Additionally, it is possible to provide specific recommendations for the development of medical and healthcare facilities in Tibet.

**Methods:**

The health scores of the participants were based on their responses to the four main sections of the questionnaire: dietary habits, living habits, health knowledge, and clinical disease history, and the variability of health status among groups with different characteristics was analyzed based on the scores. The four major sections were used to create classes of participants using latent class analysis (LCA). Using logistic regression, the factors influencing the classification of latent classes of health status were investigated.

**Results:**

A total of 995 residents from 10 counties in Naqu were selected as the study subjects. And their demographic characteristics were described. The mean health score of residents after standardization was 81.59 ± 4.68. With the exception of gender, health scores differed between groups by age, education level, different occupations, marital status, and monthly income. The health status in Naqu, Tibet, was divided into two groups (entropy = 0.29, BLRT = 0.001, LMRT = 0.001) defined as the “good health group” and the “general health group.” A monthly income of more than ¥5000 adverse to good health in Naqu, Tibet.

**Discussion:**

Single, well-educated young adults in Naqu, Tibet, have outstanding health. The vast majority of people in Tibet’s Naqu region were in good health. Furthermore, the population’s latent health status was divided into two classes, each with good dietary and living habits choices, low health knowledge, and a history of several clinical diseases. Univariate and multivariate logistic regression analysis showed that monthly income more than ¥5000 was an independent risk factor for poor health status.

## 1. Introduction

Naqu is located in the northern part of Tibet Autonomous Region, at an elevation of more than 4,500 meters. People in this area have long been subjected to the harsh environment of hypobaric hypoxia, high altitude, low temperature, and high UV rays (1). Because of its high altitude and harsh weather, Tibet is inaccessible, and its social and economic development lags behind that of the rest of China. With the development of China’s economy and transportation system in recent years, Tibetan people’s living conditions have improved to some extent. However, Tibet’s medical and health levels remain lower than in other developed areas of China. There have been few surveys and analyses of the Tibetan population’s health so far.

Currently, knowledge-attitude-practice (KAP) questions are used in the majority of population health surveys undertaken by local and international experts. Some researches use the content of questionnaires to evaluate participants’ responses and examine their health condition based on their results. Despite the fact that there are more surveys on population health status in the United States and elsewhere, there is no study of population health status in Naqu, Tibet. In addition to the traditional use of descriptive statistical analysis, several research have added latent class analysis, which is widespread in psychology and sociology, into the study of health status analysis (2–4). Our study assessed the present health status of the population in Naqu, Tibet using descriptive statistical analysis. The latent classes of health status in Naqu, Tibet were analyzed by merging the idea of health quotient (HQ) introduced by Canadian medical expert Dr.Wah Jun Tze. The health quotient can correctly reflect a person’s health wisdom and can be used to gauge a person’s attitude toward health (5). It consists of the following five components: self-care, health knowledge, living habits, mental health, and life skills. The term “health quotient” focuses on health and encompasses a wide range of topics such as culture and awareness (6). The latent class analysis of the health status of the population in Naqu of Tibet, when combined with the five elements of the health quotient, can more comprehensively show the health characteristics of the population in Naqu of Tibet.

At the National Health and Health Conference in 2016, China presented the “Healthy China 2030” plan, elevating the construction of “Healthy China” to a national strategy. Health has emerged as a “key word” in China’s future development strategy (7). The development of all aspects of society is connected to population health. Through the survey and analysis of the current health condition of the population in Naqu, Tibet, and the provision of targeted suggestions for the construction of medical and health care in Tibet, the investigation and analysis of the health status of the population here can provide data support for improving the health level of the population.

## 2. Materials and methods

### 2.1. Subjects

This was a cross-sectional survey, with data collected via questionnaires. As the study object, residents from 10 counties in Naqu, Tibet, were selected at random using the random sampling method. Following the principle of informed consent, qualified investigators conducted a face-to-face survey in the local language using a structured questionnaire. All participants of the study provided signed informed consent.

Subject inclusion criteria were as follows: (1) residents of Naqu, Tibet; (2) adults over 18 years of age; (3) good mental status and able to cooperate with the investigators to complete the questionnaire; (4) able to complete the physical index measurement.

Subjects were excluded based on the following criteria: (1) refusing to continue participating or collaborating in the process; (2) refusal to give informed consent.

The following formula was used to calculate the sample size of the study subjects:

n=tα2δ2p1−p

*n*: The minimum sample size required; *p*: The estimated proportion of the target volume; δ: maximum allowable error.

The sample size is 897 when *α* = 0.05, *p* = 0.3. In previous studies, the allowable error was generally 5% (8, 9). However, the subjects of our study were residents in plateau areas, and their occupation and physical indicators may be limited by regional influence. Therefore, δ was taken to 3% in our study in the hope of achieving a more accurate description of the health status of the inhabitants of this area.

995 people were finally determined and collected based on the calculation of the sample size of the research object and the inclusion and exclusion criteria, and all 995 people were investigated using the Questionnaire on the Health Status and Health Needs of People in Highland Areas.

### 2.2. Content of the questionnaire

1. We used a validated questionnaire on Highland People’s Health Status and Health Needs (10). The questionnaire was divided into five sections: “basic personal information,” “dietary habits,” “living habits,” “health knowledge” and “clinical disease history.”

According to the previous research, the degree to which each option represented in the sections “dietary habits,” “living habits” and “health knowledge” was assigned a score of 1–5 (Supplementary material 1). No related diseases were given 5 points in the section on “history of clinical diseases,” and the number of related diseases was taken into account when deducting the corresponding score (1–5 points) from the maximum score of 5. People who had not had any common illnesses in the previous 2 weeks receive 5 points. The maximum score of 5 points was deducted from the appropriate number based on the number of common diseases they have experienced (1–5). Those with no recent hospitalization experience received 10 points, while those with hospitalization experience received the corresponding number (1–10 points) subtracted from the maximum score of 10 points based on the number of hospitalization experiences.

To obtain a health score for the residents of the area, scores were calculated based on the responses of the participants. With a maximum score of 133 points and a maximum score of 100 points after score standardization, higher scores represent better health.

1. According to some questions in “health knowledge,” the current situation and demand of local residents for daily physical examination, disease screening activities and health promotion activities were analyzed, and the influence of health status score on health knowledge was analyzed.

### 2.3. Questionnaire quality control

1. The first draft of the questionnaire was created after referring to a large number of research materials, and then revised after contacting experts and conducting a pre-survey before forming the final questionnaire for this survey and creating a survey guide.
2. The questionnaire’s content was kept confidential to avoid respondents from not filling out the actual scenario out of fear of personal information leakage.

### 2.4. Statistical analysis

A database was created using the EpiData3.1 program. The data was sorted using Excel2016, and the statistical analysis was performed using SPSS26.0 software. Values for regularly distributed measurement data and categorical variables are expressed as mean standard deviation (SD) and number/percentage (*n*/%). The *t*-test and ANOVA were used to describe differences in participant characteristics. All variables were included in univariate and multivariate logistic regression models for analysis. The difference was considered statistically significant when *p* < 0.05 and the test level *α* = 0.05. The representative meaning of categories was investigated using latent class analysis (LCA), which was used to establish classes of participants based on their answers to the four primary sections. The explicit variables of latent class analysis were dietary habits score, living habits score, health knowledge score and clinical disease history score. Before the standardized score, a dietary habits score ≥ 23 was defined as having good dietary habits. The score of living habits ≥19 was defined as having good living habits. Good health knowledge was defined as health knowledge score ≥ 10; the clinical disease history score ≥ 55 was defined as no clinical disease history. Before performing latent class analysis, univariate and multivariate logistic regression analyses were performed in SPSS26.0 software to compare the scores of the four sections. The high and low score groups were divided according to the above. Models with 1–4 classes were examined, and model selection was based on improvements in test statistics including chi-square goodness of fit, Bayesian information criterion (BIC), Akaike information criterion (AIC), entropy, Bootstrap likelihood ratio test (BLRT) and Vuong-lo–mendell–rubin test (LMRT). Mplus8.3 software was used to do the Latent class analysis. In SPSS26.0 software, logistic regression was utilized to investigate the contributing factors of latent classes of health status.

## 3. Results

### 3.1. Participant characteristics

This study collected a total of 995 valid questionnaires (Table 1), including 586 males (58.9%). There were 409 females, accounting for 41.1% of the total. The average age was 34.16 ± 13.04 years old. 54.5% were illiterate, 20.2% had completed primary or junior high school, and 25.3% had completed high school or higher. And herders and farmers made up 63.3% of the participants. Furthermore, in this study, 70.1% were married.

**Table 1:** Participant characteristic.

Characteristic	*n* (%)	Mean total score (Mean ± SD)	Value of statistics	*p* value
Gender				
Male	586 (58.9)	81.69 ± 4.69	0.85	0.397
Female	409 (41.1)	81.44 ± 4.67		
Age groups, years				
18 ~ 30	491 (49.3)	82.16 ± 4.81	10.57	<0.001
31 ~ 50	381 (38.3)	81.33 ± 4.38		
51~	123 (12.4)	80.10 ± 4.70		
Education				
Illiteracy	542 (54.5)	81.03 ± 4.39	21.97	<0.001
Primary and Junior High schools	201 (20.2)	81.01 ± 4.72		
High school and above	252 (25.3)	83.24 ± 4.88		
Occupations				
Herdsmen and farmer	630 (63.3)	80.82 ± 4.63	−6.95	<0.001
other	365 (36.7)	84.12 ± 4.52		
Marital status				
Married	697 (70.1)	81.13 ± 4.38	−4.42	<0.001
Unmarried	298 (29.9)	82.65 ± 5.16		
Monthly income (RMB)				
Below 2000	312 (31.4)	81.15 ± 4.95	4.00	0.019
2001 ~ 5,000	358 (36.0)	82.13 ± 4.69		
More than 5,000	325 (32.7)	81.41 ± 4.68		

### 3.2. Differences in total health scores between groups of people with various characteristics

The health of the population was graded based on the questionnaire responses of the participants. Following standardization, the participants’ health status could be assessed up to 100 points, with a higher score indicating better health.

We used *t*-test and ANOVA to compare the health scores of people of different genders, ages, education levels, occupations, marital status, and monthly income (Table 1). And the results revealed that there were differences in the total health score in terms of age, education, occupations, marriage, and monthly income in addition to gender. The higher the health score, the younger the age. People with a high school education or higher had a considerably higher health score (83.24 ± 4.88) than those with lower education levels. Herdsmen’s and farmers’ health scores (80.82 ± 4.63) were lower than other occupations. Unmarried people had a higher health score (82.65 ± 5.16) than who were married. People with a monthly income of ¥2001–5,000 could have a higher health score.

### 3.3. Differences in the four sections’ health scores between groups of persons with various characteristics

Following the comparison of the overall score, the standardized scores for the different sections were compared (Table 2).

**Table 2:** Comparison of average scores of health scores in each section.

Characteristic	Dietary habits	*p*	Living habits	*p*	Health knowledge	*p*	Clinical disease history	*p*
Gender								
Male	19.21 ± 1.80	0.736	16.71 ± 2.01	0.001	6.25 ± 1.61	0.293	39.52 ± 2.15	<0.001
Female	19.25 ± 1.64		17.11 ± 1.86		6.14 ± 1.69		39.28 ± 2.36	
Age groups, years								
18 ~ 30	19.17 ± 1.86	0.474	17.14 ± 2.06	<0.001	6.31 ± 1.68	0.138	39.53 ± 2.11	<0.001
31 ~ 50	19.31 ± 1.59		16.62 ± 1.79		6.12 ± 1.61		39.28 ± 2.37	
51~	19.19 ± 1.74		16.58 ± 1.88		6.08 ± 1.57		38.25 ± 2.98	
Education								
Illiteracy	19.30 ± 1.58	<0.001	16.57 ± 1.86	<0.028	6.00 ± 1.55	<0.001	39.16 ± 2.51	0.070
Primary and Junior High schools	18.94 ± 1.94		16.69 ± 1.85		6.15 ± 1.64		39.24 ± 2.15	
High school and above	19.30 ± 1.85		17.66 ± 2.02		6.71 ± 1.73		39.57 ± 2.19	
Occupations								
Herdsmen and farmer	19.10 ± 1.74	0.003	16.63 ± 1.86	<0.001	5.91 ± 1.59	<0.001	39.18 ± 2.46	0.093
other	19.44 ± 1.71		17.29 ± 2.04		6.73 ± 1.60		39.44 ± 2.18	
Marital status								
Married	19.34 ± 1.57	0.005	16.57 ± 1.86	<0.001	6.10 ± 1.60	0.003	39.12 ± 2.39	0.001
Unmarried	18.96 ± 2.04		17.58 ± 1.98		6.45 ± 1.72		39.64 ± 2.27	
Monthly income (RMB)								
Below 2000	19.31 ± 1.61	0.020	16.71 ± 1.91	0.063	5.94 ± 1.69	0.001	39.19 ± 2.57	0.252
2001 ~ 5,000	19.36 ± 1.69		17.06 ± 2.07		6.27 ± 1.60		39.44 ± 2.32	
More than 5,000	19.01 ± 1.88		16.82 ± 1.86		6.40 ± 1.61		39.18 ± 2.19	

In terms of dietary habits, we questioned about Eating on time; Eating speed; The temperature of food; A few meals a day; The number of breakfast times per week; How many times a week you eat leftovers; Whether the food has a strong sour taste; How to deal with moldy ingredients. There were differences among groups with different education levels, different occupations, marital status and monthly income. Those with primary and junior high school education performed worse than those with other qualifications. The scores of dietary habits of herdsmen and farmers were significantly lower than other occupations. The score of dietary habits of married group was significantly higher than that of the unmarried groups. People with monthly income from ¥2001 to ¥5,000 had higher dietary habits scores than those with other income levels. Univariate logistic regression analysis of dietary habits showed that education level of primary and junior high school and being unmarried were associated with lower scores in this section (Table 3). On the basis of single factor regression analysis, the multivariate logistic regression further found that other occupations were 2.01 times more likely to get high scores in terms of dietary habits than herdsmen and farmers.

**Table 3:** Univariate and multivariate logistic regression analysis of the scores of each section.

Characteristic	Dietary habits				Living habits				Health knowledge				Clinical disease history			
	Crude		Adjusted		Crude		Adjusted		Crude		Adjusted		Crude		Adjusted	
	OR (95%CI)	*p*	OR (95%CI)	*p*	OR (95%CI)	*p*	OR (95%CI)	*p*	OR (95%CI)	*p*	OR (95%CI)	*p*	OR (95%CI)	*p*	OR (95%CI)	*p*
Gender																
Male																
Female	1.03 (0.677–1.576)	0.881	1.13 (0.721–1.762)	0.600	2.24 (1.232–4.065)	0.008	2.14 (1.156–3.950)	0.015	0.85 (0.636–1.137)	0.274	0.80 (0.588–1.090)	0.158	0.77 (0.572–1.042)	0.091	0.74 (0.538–1.009)	0.057
Age groups, years																
18 ~ 30																
31 ~ 50	1.34 (0.839–2.131)	0.222	0.99 (0.557–1.745)	0.961	0.80 (0.452–1.402)	0.430	1.22 (0.645–2.318)	0.538	0.79 (0.585–1.080)	0.142	1.11 (0.759–1.628)	0.586	0.96 (0.701–1.303)	0.775	1.20 (0.816–1.755)	0.359
51~	0.79 (0.436–1.442)	0.446	0.57 (0.286–1.135)	0.109	0.63 (0.296–1.348)	0.235	1.93(0.412–2.090)	0.856	0.78 (0.493–1.28)	0.293	1.18 (0.707–1.983)	0.521	0.64 (0.390–1.067)	0.088	0.76 (0.438–1.324)	0.335
Education																
Illiteracy																
Primary and Junior High schools	0.52 (0.318–0.864)	0.011	0.60 (0.348–1.043)	0.070	1.01 (0.534–1.900)	0.983	1.05 (0.535–2.045)	0.894	1.41 (0.969–2.051)	0.072	1.38 (0.924–2.054)	0.116	0.83 (0.558–1.232)	0.353	0.77 (0.505–1.185)	0.24
High school and above	0.77 (0.462–1.279)	0.310	0.95 (0.443–2.031)	0.892	2.03 (0.969–4.274)	0.061	1.97 (0.740–5.242)	0.175	2.24 (1.61–3.119)	<0.001	1.78 (1.093–2.886)	0.02	1.24 (0.886–1.746)	0.208	1.00 (0.596–1.665)	0.99
Occupations																
Herdsmen and farmer																
Other	1.24 (0.796–1.927)	0.344	2.02 (1.118–3.634)	0.020	1.03 (0.600–1.764)	0.918	1.61 (0.319–1.171)	0.138	2.29 (1.716–3.058)	<0.001	1.93 (1.337–2.795)	<0.001	1.19 (0.889–1.600)	0.26	0.93 (0.630–1.384)	0.734
Marital status																
Married																
Unmarried	0.47 (0.310–0.720)	<0.001	0.34 (0.200–0.575)	<0.001	2.02 (1.034–3.927)	0.040	1.68 (0.757–3.750)	0.202	1.49 (1.100–2.005)	0.009	0.91 (0.610–1.348)	0.628	1.80 (1.324–2.436)	<0.001	2.06 (1.387–3.070)	<0.001
Monthly income (RMB)																
Below 2000																
2001 ~ 5,000	1.21 (0.690–2.120)	0.506	1.19 (0.670–2.119)	0.551	1.67 (0.881–3.170)	0.116	1.93 (0.993–3.756)	0.053	1.15 (0.806–1.627)	0.450	0.99 (0.687–1.428)	0.960	1.30 (0.918–1.837)	0.140	1.20 (0.841–1.722)	0.311
More than 5,000	0.58 (0.348–0.951)	0.031	0.53 (0.310–0.890)	0.017	1.27 (0.687–2.349)	0.445	1.32 (0.679–2.552)	0.415	1.25 (0.876–1.785)	0.218	0.91 (0.623–1.335)	0.636	0.76 (0.519–1.109)	0.154	0.70 (0.471–1.045)	0.081

In terms of living habits, we inquired about the following: The quality of sleep; Whether you have the habit of napping; Whether there is a habit of drinking more water; Smoking status; Secondhand smoke exposure in the last year; Alcohol consumption; Do you usually do physical exercise? There were differences between groups with different genders, ages, education levels, different occupations and marital status. Female’s living habits are better than males. Univariate and multivariate logistic regression analysis showed that women were more than twice as likely to have better lifestyle habits than men (Table 3). In addition, the younger the age, the higher the average score of living habits. The higher the level of education, the higher the average living habits score. Herdsmen and farmers score lower than other occupations on living habits. And the unmarried group outperformed the married groups in terms of living habits.

We questioned health knowledge by asking Whether visiting the hospital regularly for physical examination; Whether participated in disease screening activities; Whether participated in health education activities; Knowledge of health; Psychological knowledge. There were differences in average health knowledge scores across education levels, different occupations, marital status, and monthly income categories. The higher the level of education, the higher the average health knowledge score. Herdsmen and farmers had a lower health knowledge score than the other occupations. Unmarried people had higher health knowledge scores than married people. The higher the monthly income, the higher the level of health knowledge. Logistic regression analysis showed that high school and above education and being unmarried increased the likelihood of getting high scores in the health knowledge part, while being a herdsman or farmer reduced the likelihood of getting high scores. However, the effect of being unmarried on health knowledge was not significant in the results of multifactor logistic regression analysis (Table 3).

In terms of clinical history, we asked whether were any hypoxic symptoms such as tinnitus, dizziness, limb weakness, and shortness of breath. History of intestinal disease, cardiovascular disease, cerebrovascular disease, blood and stomach disease, lung disease, altitude disease, cancer; Whether they suffered from other common diseases in the past 2 weeks; The number of hospitalizations in the last 2 weeks. Gender, age, and marital status all differed significantly between groups. Male clinical disease history scores were higher than female clinical disease history scores. The older the person, the lower the clinical disease history score. The unmarried group outperformed the married groups in terms of clinical disease history. Both univariate and multivariate logistic regression analyses showed less clinical disease history among unmarried people. And more clinical disease history among those with a monthly income of more than ¥5,000 in multivariate logistic regression analysis.

### 3.4. Health knowledge analysis

**Figure 1:**
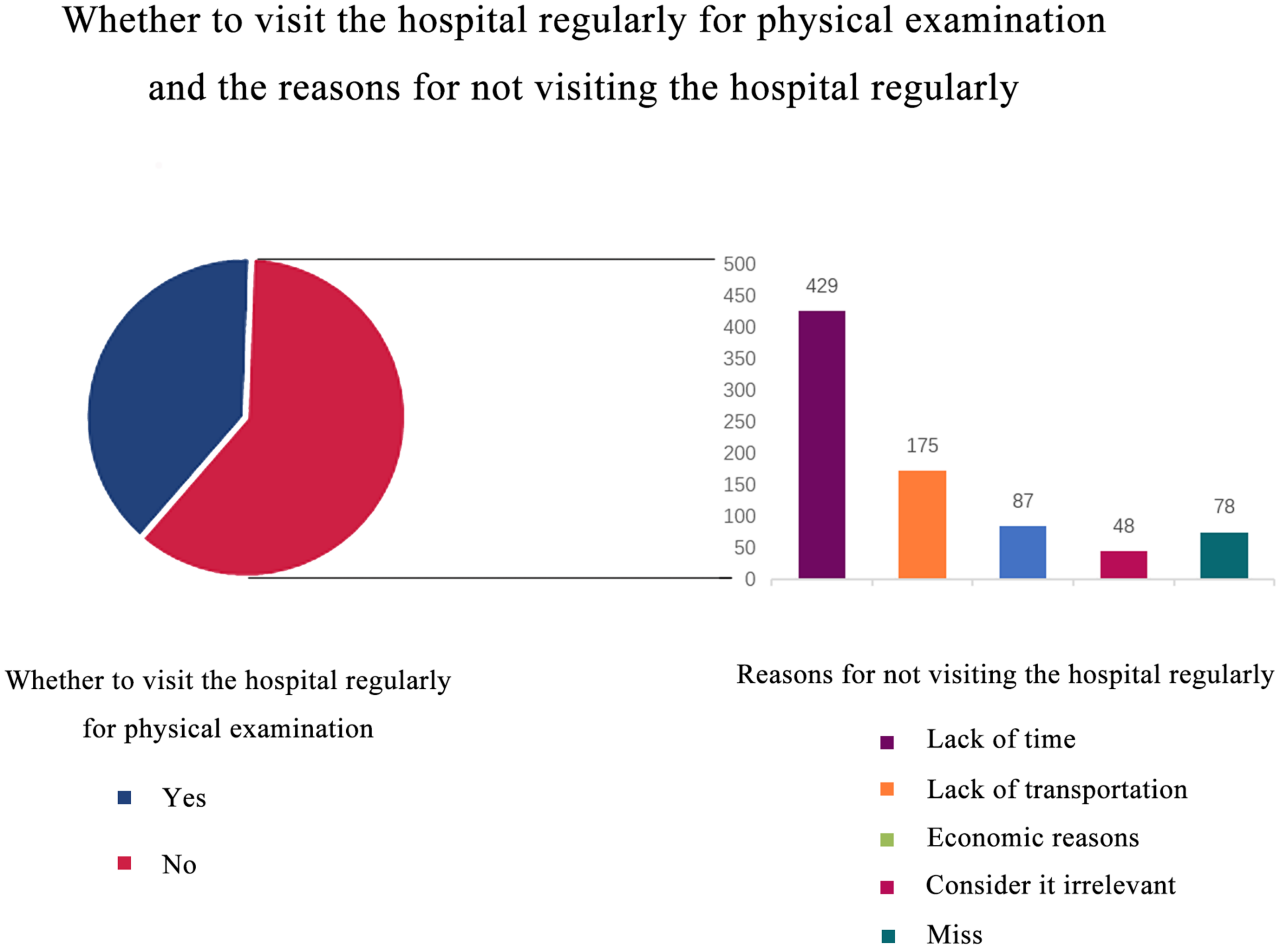
Whether to visit the hospital regularly for physical examination and the reasons for not visiting the hospital regularly. 39.2% of them would go to the hospital regularly for physical examination, while 60.8% of them would not. We used the bar chart to analyze the reasons for not going to the hospital regularly, and the most common reason was lack of time.

In addition, a separate analysis of the health knowledge section showed that 390 (39.2%) of the population visited the hospital regularly for physical examination, while 605 did not. There were 527 people who responded to the question about why they did not visit the hospital regularly. Of the 527 respondents, 429 cited lacks of time as a cause, 175 cited continual transit as a reason, 48 deemed regular physical examination unnecessary, and 87 cited economic reasons for not having regular health examinations (Figure 1).

**Figure 2:**
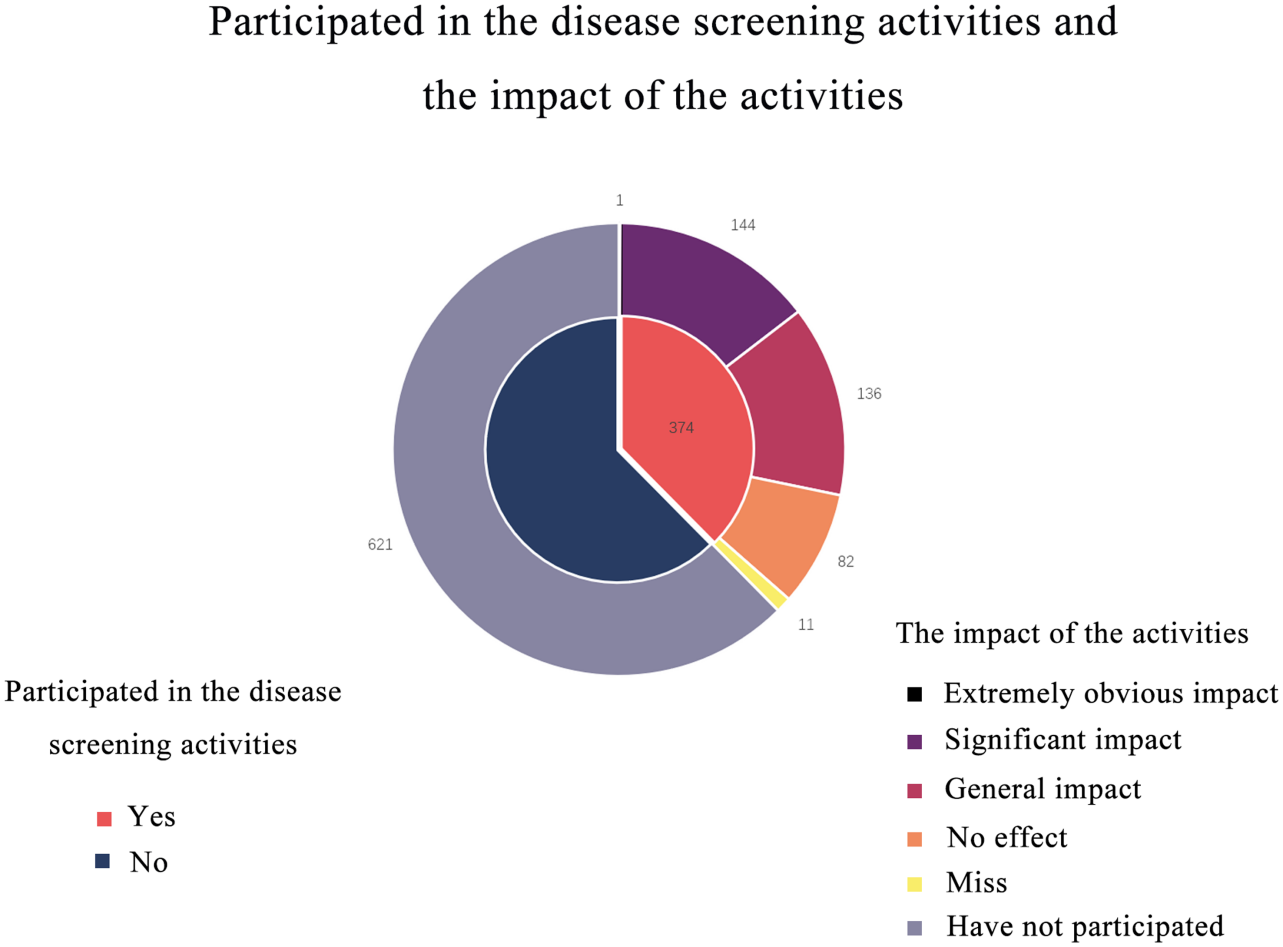
Double-layer pie chart shows participation in screening activities and the impact of the activities. 374 (37.6%) people participated in disease screening activities. Most people believed that the screening program had an impact on their health behavior.

374 (37.6%) people participated in disease screening activities. A total of 363 people answered the question whether participating in disease screening activities would affect their health behavior, and 82 people thought that it had no effect. 136 thought that the impact was general; 144 thought that the impact was significant; One person thought that the impact was extremely obvious (Figure 2).

278 (27.9%) of the participants had participated in health education activities. 275 people answered the question of the access to participate in health education activities, 69 people took the hospital as the channel; 87 used community as the channel; 64 of them had their work unit as the channel; 35 people use internet media such as television and radio as channels. Among the questions about the number of times they participated in health education activities in the past 2 years, 63 people had participated one or two times; 40 people participated 3–5 times; 10 participated in more than five health promotion sessions. 96 people thought that health promotion activities did not affect their health behavior; 97 people thought that participating in health activities had a general effect on their health behavior, 71 people thought that participating in health promotion activities had a significant effect on their health behavior. Three people thought that the influence on themselves was very obvious.

**Figure 3:**
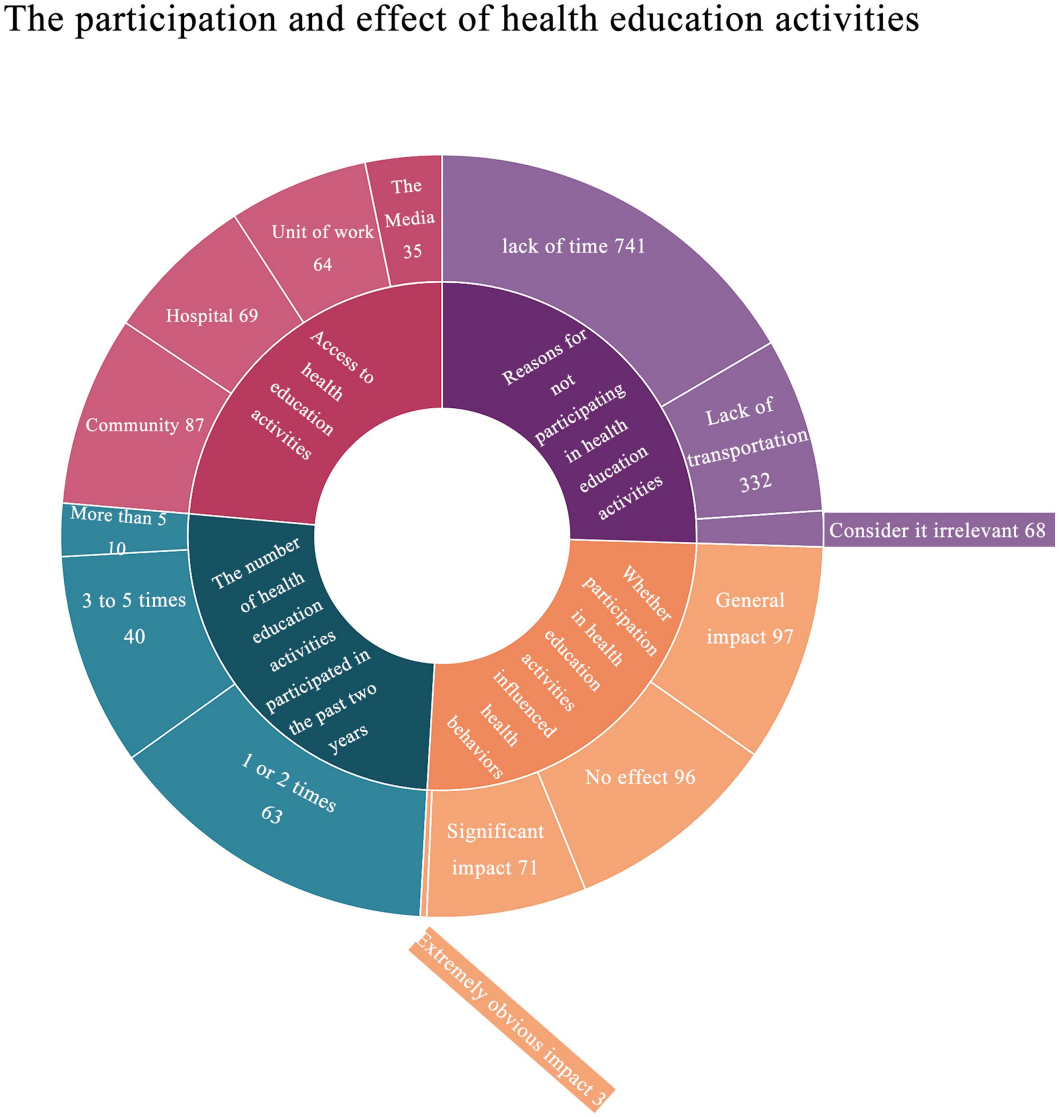
The participation and effect of health education activities. Each question accounted for an average of one quarter of the inner circle, and the corresponding response to each question was distributed according to the proportion within the inner quarter circle.

741 people did not participate in health education activities due to a lack of time; 332 people did not participate in health education activities due to inconvenient transportation; and 68 people did not participate in health education activities because they believed the content was irrelevant to them (Figure 3). 275 persons were able to master three or more types of health knowledge; 207 people were able to master the knowledge of high altitude disease prevention and control, as well as high altitude environment and health care. 772 persons could master two or more types of mental health knowledge, and 161 could master mental response and adaptation at high altitude knowledge.

### 3.5. Latent class analysis of population health status

Based on the five elements of self-care, health knowledge, living habits, mental health, and life skills of “health quotient,” combined with the corresponding items in the questionnaire, dietary habits, living habits, health knowledge, and clinical disease history were taken as the explicit variables, which were divided into 1 to 4 classes in order for model fitting and estimation of latent class analysis to classify the potential types of health status of the population in Naqu, Tibet. The relationship between latent health categories and gender, age, different occupations, education level, marital status and monthly income were analyzed.

**Figure 4:**
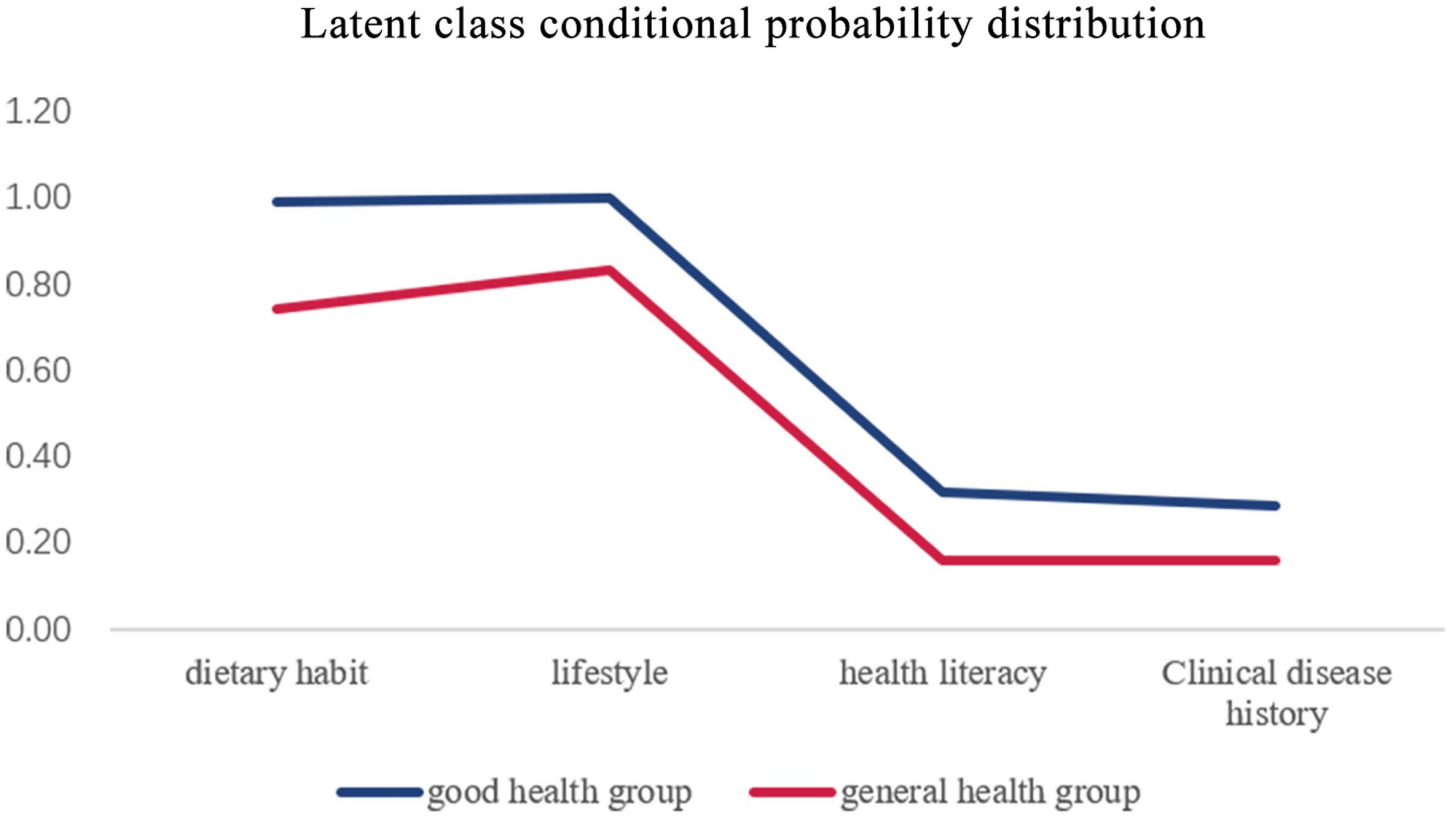
Latent class conditional probability distribution. Latent class analysis divided the population into two groups: good health group and general health group. The line graph shows how high or low each group scored on the four dimensions, representing each type of feature.

The results revealed that the AIC, BIC, and aBIC values of the two-class model are the smallest (Table 4), while the Entropy value was the highest, and LMRT and BLRT both approach the significant threshold. As a result, the two-class model was chosen as the best model, and the conditional probability distributions for each class were illustrated in Figure 4; Table 5. The results showed that the likelihood of the four characteristics of category 1 was higher than that of category 2, therefore they were dubbed the “good health group,” with a total of 851 cases (86.0%) being assigned to this category. Category 2 was dubbed the “general health group,” with 144 cases (14.0%) falling into this category. The two latent classes also shared several characteristics: high scores in dietary habits and living habits, and low scores in both.

**Table 4:** Latent class model fitting index of health status.

Model	K	Log(L)	AIC	BIC	aBIC	Entropy	BLRT(*p*)	LMRT(*p*)
1	4	−2615.82	5239.65	5259.26	5246.56			
2	9	−1656.51	3331.03	3375.15	3346.57	0.29	<0.001	<0.001
3	14	−1654.98	3337.95	3406.59	3362.13	0.63	0.67	0.36
4	19	−1654.27	3346.55	3439.70	3379.35	0.69	0.67	0.59

**Table 5:** Latent class conditional probability distribution.

Class	*n*(%)	Dietary habits	Lifestyle	Health literacy	Clinical disease history
Good health group	851 (0.86)	0.99	1.00	0.32	0.29
General health group	144 (0.14)	0.74	0.83	0.16	0.16

### 3.6. Influencing factors of latent classes of population health status in Naqu, Tibet

Combined with the existing researches, the relationship between gender, age, education level, different occupations, marital status, and monthly income and the latent health classes of the population in Naqu area of Tibet was analyzed as independent variables. Univariate logistic regression analysis (Table 6) showed that Monthly income of more than ¥5,000 was related to the grouping of latent health classes of the population in Naqu, Tibet. After adjusting for other factors, the results of the multivariate logistic regression analysis still showed that the health of those monthly income more than ¥5,000 was poor than others (Table 6). Univariate and multivariate logistic regression analysis showed that monthly income more than ¥5,000 was an independent risk factor for poor health status.

**Table 6:** Univariate and multivariate logistic regression analysis were performed after latent class analysis.

Characteristic	Class1(%)	Class2(%)	Crude			Adjusted		
			OR	95%CI	*p*	OR	95%CI	*p*
Gender								
Male	492 (57.8)	94 (65.3)						
Female	359 (42.2)	50 (34.7)	1.37	0.948–1.983	0.093	1.43	0.975–2.103	0.067
Age groups, years								
18 ~ 30	421 (49.5)	70 (48.6)						
31 ~ 50	329 (38.7)	52 (36.1)	1.052	0.715–1.548	0.797	1.091	0.689–1.727	0.711
51~	101 (11.9)	22 (15.3)	0.764	0.451–1.292	0.315	0.759	0.423–1.357	0.351
Education								
Illiteracy	468 (55.0)	74 (51.4)						
Primary and Junior High schools	164 (19.3)	37 (25.7)	0.701	0.455–1.081	0.108	0.790	0.498–1.256	0.319
High school and above	219 (25.7)	33 (22.9)	1.050	0.676–1.630	0.830	1.287	0.682–2.43	0.437
Occupations								
Herdsmen and farmer	537 (63.1)	93 (64.6)						
Other	314 (36.9)	51 (35.4)	1.07	0.738–1.541	0.733	1.167	0.730–1.866	0.517
Marital status								
Married	601 (70.6)	96 (66.7)						
Unmarried	250(29.4)	48(33.3)	0.8321	0.571–1.212	0.338	0.676	0.423–1.080	0.102
Monthly income (RMB)								
Below 2000	271 (31.8)	41 (28.5)						
2001 ~ 5,000	318 (37.4)	40 (27.8)	1.203	0.756–1.914	0.43	1.242	0.772–1.996	0.372
More than 5,000	262 (30.8)	63 (43.8)	0.630	0.410–0.966	0.034	0.596	0.381–0.933	0.024

## 4. Discussion

The Tibetan region of China has a unique alpine climate and hypoxic environment. The duration of sunlight in Tibetan highlands is long, and UV radiation is substantially higher than in lower altitude areas. In this extreme environment, Tibetan living habits and diets are strongly influenced by regional biogeography, indigenous traditions, popular religious beliefs and dietary taboos (11, 12). It is characterized by beef and mutton meat, dairy products, and fewer fruits and vegetables, and its vitamin C intake is much lower than that in plain areas. Their daily staple food is zanba, and a common drink is buttered tea (10, 13). Therefore, the diet of high-altitude residents is high in fat, high in cholesterol, and low in vitamins (14). Plains, low oxygen stress has a major impact on the physiological functions of people who have lived on the plains for generations and have recently moved to the plateau. However, the indigenous Tibetans have a strong adaptability to the plateau (15), and it has been confirmed that this adaptability to high altitude may be related to the genes of the Tibetan people (16, 17).

It has been reported that 1.2–33% of the population at high altitude are not adapted to the hypoxic environment, and are prone to hypercellularity of high altitude polycythemia (HAPC), which results in increased blood viscosity, slow blood flow, increased blood volume, aggravated ischemia and hypoxia, microcirculatory disturbances, and affected systemic tissues and organs. Patients are prone to dizziness, headache, dyspnea, palpitations, sleep disturbances and other symptoms, and are susceptible to stroke and myocardial ischemia (18). A cross-sectional study in 2015 showed that the prevalence of hypertension, diabetes, overweight/obesity, dyslipidemia, and current smoking in Tibetan adults were 62.4, 6.4, 34.3, 42.7, and 6.1%, respectively. A previous study showed that the age-standardized prevalence rates of dyslipidemia, hypertension, diabetes, current smoking, and overweight in Chinese adults aged 35–74 years were 53.6, 26.1, 5.2, 34.4, and 28.2%, respectively, and that the prevalence rates of dyslipidemia were higher in those who did not consume butter tea (19).

The four sections included in our health score were: dietary habits, living habits, health knowledge, and clinical disease history. The *t*-test and ANOVA results of the total health score of different groups revealed that the older the age, the lower the health score; people with a high school education or higher had significantly higher health scores than those with lower education levels. Unemployed, herdsmen, and farmers had worse health scores than the general population, whereas students and other occupational categories had higher health scores than the general population. Unmarried people had better health than those in other marital status groups. The higher the monthly *per capita* income, the better the health. Single young people with higher education and higher income have higher health scores and better physical health status. The results were similar to those in other regions of China (20–22).

Taking dietary habits, living habits, health knowledge and clinical disease history as explicit variables, the potential groups of health status of the population in Naqu, Tibet were classified. The subjects were divided into two classes. The probability of the four dimensions of class 1 was higher than that of class2, so it was named as “good health group” and “general health group.” The relationship between latent health classes and gender, age, different occupations, education, marital status, monthly income was analyzed.

The results showed that the probability of four dimensions was higher in the “good health group” than in the “general health group,” and the two latent classes also shared some common characteristics: high scores in dietary habits and living habits, and low scores in both health knowledge and clinical disease history. According to univariate and multivariate logistic regression analysis, monthly income higher than 5,000 was an independent risk factor for influencing the study participants to be classified in the group with poor health status.

The total score did not differ significantly between genders, although there were significant difference in living habits and clinical disease history. Women’s living habits scores were higher than men’s, but their clinical disease history scores were lower. Gender has been identified as a major factor influencing living habits (23). In addition, according to the 2018 National Health Service Survey, the two-week prevalence rate of women in Tibet was much greater than that of men. The two-week prevalence rate was positively related to age and economic level, but negatively related to education (24). The health of Tibetan women demands our concern due to their physiological constitution and the stress of living.

At the same time, differences in health scores between age groups in terms of living habits and clinical disease history were statistically significant. The younger the age, the healthier the living habits and the fewer clinical disease history. This could be due to the fact that they live less stressful lives, have better sleep habits, can maintain exercise habits, and may smoke and drink less. The older you get, the worse your physical function becomes and the more clinical disease history you have. In addition to our conventional notions of undesirable habits such as smoking and drinking. In recent years, an increasing number of studies have discovered that sleep quality is closely associated to health. Sleep difficulties have been linked to inflammatory responses in the body, psychological issues, and even increased mortality in adults (25–27). As a result, excellent sleep habits, regular exercise, and quitting smoking and drinking can all help to improve physical health.

In terms of dietary habits, living habits, and health knowledge, our study discovered that differences in health scores of different education levels and different occupations were statistically significant. There were also statistical differences in clinical disease history scores between different occupations. The higher the educational level, the higher the social position, the more the emphasis on health, the greater the social contact, and the more hopeful mental state, which influences living habits (28). According to research studies, highly educated people have a high level of health information literacy (29). As for the fact that people with primary and junior high school education have lower dietary habits than the other two groups, we speculate that this is because people with this education level are mostly herdsmen and farmers in the Naqu. They cannot ensure a regular three meals a day during their occupations.

Animal husbandry is the pillar industry of Tibet, and the Tibetan people have a profound historical tradition and rich practical experience in animal husbandry since ancient times (30). 63.3% of our participants were herders or farmers. Herdsmen’s and farmers eat fewer fresh vegetables and fruits all year because of their nomadic living habits, and herders and farmers’ domicile and access to medical institutions could be better (24). And they have limited access to health care and a poor understanding of health information (31). As a result, herders and farmers performed poorly in all four components. Students performed better in all four parts, most likely because they were younger, had a normal school schedule, ate three meals per day, and were given more health information. Thus, herdsmen and farmers performed poorly in all four sections.

In all four sections, the differences in health scores by marital status were statistically significant. Marriage can greatly increase income and happiness, as well as promote physical and mental health. Marriage also brings greater happiness to men than to women (32, 33). However, the results of our *t*-test, ANOVA, logistic regression analysis, and subsequent analysis of latent categories showed that the unmarried group scored lower than the married group only in terms of dietary habits. Married people are more likely to have a regular family life and therefore better dietary habits than the unmarried group. Unmarried people had a higher average living habits score than the married group, probably because their lives were freer and more flexible. Although the results of some studies are contrary to our results, according to our survey on the living conditions of local residents, the married people may bear more social, family and work pressure than the unmarried people. These hidden pressures cannot be revealed through existing indicators. Our study also did not examine life well-being, which may be one of the reasons why unmarried people in this paper have better health than married people.

People with a monthly income of between ¥2001 and ¥5,000 were considered to be well-off, as they could afford three meals a day and regular work and rest, and their life pressures were moderate, so their dietary habits score was higher than other income groups. As a result, their dietary habits, living habits and clinical disease history scores were higher than those of other income groups. The higher the level of education, the higher the health knowledge score; and the higher the monthly *per capita* income, the higher the health knowledge score. People with higher monthly incomes pay more attention to health issues and therefore have more comprehensive health and psychological knowledge. Higher monthly income contributes to the acceptance of health knowledge (34), but the work stress and adverse health outcomes associated with higher income cannot be ignored (35).

In a separate analysis of health information literacy, it is not difficult to see that many people are in the habit of having frequent physical examinations at the hospital, as well as participating in disease screening and health promotion activities. However, transport and income constraints prevent people from having regular health checks. According to the results of our study, more than half of the participants believed that “whether participating in health screening activities will affect their health behavior” and “whether participating in health promotion activities will affect their health behavior” had a moderate or no effect on their health behavior.

On the one hand, we can find answers to the health education channels; however, the intrinsic education activities through community and hospital channels are unable to attract more people to participate in the form of a single, and its influence is also unimpressive; on the other hand, the educational level of our subjects is relatively low; the majority of them are illiterate or have just completed primary and junior high school. According to a 2021 study conducted in Nanjing, China, current health education and promotion intervention tactics are ineffective for people with low education levels (29). As a result, it is essential to develop diverse and highly targeted personalized health education approaches to reduce the difficulty of absorbing health education information among low-educated groups.

In general, the people of Naqu, Tibet, have healthy dietary habits and living habits, but they have lower health knowledge and a higher prevalence of clinical diseases. Disease screening and health education should be improved. Tibet’s economy has grown dramatically in recent years, and living conditions have improved.

However, there is still a need to continue to improve the level of medical and health care in the Naqu, with particular attention to the health level of herders and farmers and the dissemination of health knowledge for this population, introduce medical and health professionals, provide free medical care, and carry out other activities to promote the health of people in Naqu.

## 5. Conclusion

Single, well-educated young adults in Naqu, Tibet, have outstanding physical and mental health. The vast majority of people in Tibet’s Naqu region were in good health. Furthermore, the population’s latent health status was divided into two classes, each with good dietary and living habits choices, low health knowledge, and a history of several clinical diseases. Univariate and multivariate logistic regression analysis showed that monthly income more than ¥5,000 was an independent risk factor for poor health status.

## 6. Limitations

There are several limitations that must be addressed. This is a cross-sectional study, so the conclusion of causality is weak. Alternatively, some of the items in our questionnaire may cause respondents to have a recall bias. Our judgments of the participants’ health were based on past research and conventional knowledge, which may have been skewed.

## Data availability statement

The raw data supporting the conclusions of this article will be made available by the authors, without undue reservation.

## Ethics statement

The studies involving humans were approved by Naqu People’s Hospital Medical research and New technology Ethics Committee. The studies were conducted in accordance with the local legislation and institutional requirements. The participants provided their written informed consent to participate in this study.

## Author contributions

JC: original draft preparation, methodology, software analysis, formal analysis, and data curation. ON: investigation, resources, manuscript review, and editing. DZ, CJ, RW, YC, and YD: investigation, data entry, and data curation. XX and XL: investigation, resources, manuscript review, editing, and supervision. All authors contributed to the article and approved the submitted version.
