# Supplementary material for: Health status of the population in Naqu, Tibet and its latent class analysis: a cross-sectional survey
Source: Front Public Health. 2023 Nov 3;11:1223382. doi: 10.3389/fpubh.2023.1223382 (PMC10654740; doi:10.3389/fpubh.2023.1223382)
Supplement: Supplementary file 1 [file Table_1.DOCX]

Supplementary Material

Health Status of the Population in Naqu, Tibet and its Latent Class Analysis: a cross-sectional survey

Jiaxue Cui^1†^, Ouzhu Nima^2†^, Duoji Zhaxi^2^, Chenxin Jin^1^, Ruiqi Wang^1^, Yizhuo Diao^1^, Yongxing Chen^1^, Xiaoguang Xu^2,3*^, Xiaofeng Li^1*^

*** Correspondence:**Xiaofeng Li

lxf_chen@dmu.edu.cn

Xiaoguang Xu

xuxg2007@sina.com

# Supplementary Tables

Table 1 According to the previous research, the degree to which each option represented in the sections “dietary habits”, “living habits” and “health knowledge” was assigned a score of 1-5.

| **Section** | **Questions** | **Criterion for scoring** | | | | | | | |
| --- | --- | --- | --- | --- | --- | --- | --- | --- | --- |
| Dietary habit | Eating on time | Not on time =1 | | Occasionally on time =2 | | Largely on time =3 | | strictly on time =4 | |
|  | Eating speed | Very fast =1 | | Fast =2 | | Slow =3 | | Average =4 | |
|  | The temperature of food | Cold =1 | | Very hot =2 | | Hot =3 | | Warm =4 | |
|  | A few meals a day | meal =1 | | 2meal =2, | | 4meal =3 | | 3meal =4 | |
|  | The number of breakfast times per week | Less than 3 days =1 | | 3-4 days =2 | | 5-6 days =3 | | 7 days =4 | |
|  | How many times a week you eat leftovers | More than 5 times =1 | | 3-4 times =2 | | 1-2 times =3 | | 0 times =4 | |
|  | Whether the food has a strong sour taste | Always =1 | | Often =2 | | occasionally =3 | | Never =4 | |
|  | How to deal with moldy ingredients | Eat most of the ingredients =1 | | Do not expose the food to the sun and throw away the moldy parts =2 | | Expose the ingredients to the sun and throw away the moldy parts =3 | | Throw away completely =4 | |
| Living habits | the quality of sleep | Very poor =1 | Poor =2 | | Generally good =3 | | Good =4 | | Very good =5 |
|  | Whether you have the habit of napping | Never =1 | Occasionally =2 | | General =3 | | Often =4 | | Always =5 |
|  | Whether there is a habit of drinking more water | Never =1 | Occasionally =2 | | General =3 | | Often =4 | | Always =5 |
|  | Smoking status | Former smoker and current smoker =1 | Former smoker and now successfully quit =2 | | Never =3 | |  | |  |
|  | Secondhand smoke exposure in the last year | More than 6 days/week =1 | 5-6 days/week =2 | | 3-4 days/week =3 | | 1-2 days/week =4 | | Basically none =5 |
|  | Alcohol consumption | Used to drink and still drinks =1 | Used to drink and is now successfully abstinent =2 | | Never =3 | |  | |  |
|  | Do you usually do physical exercise? | Never =1 | Occasionally =2 | | General =3 | | Often =4 | | Always =5 |
| Health knowledge | whether visit the hospital regularly for physical examination | No=1 | Yes=2 | |  | |  | |  |
|  | Whether they have participated in disease screening activities | No=1 | Yes=2 | |  | |  | |  |
|  | Whether they have participated in health education activities | No=1 | Yes=2 | |  | |  | |  |
|  | Knowledge of health | One point is added for each item mastered, up to a maximum of 5 points |  | |  | |  | |  |
|  | Psychological knowledge | One point is added for each item mastered, up to a maximum of 4 points |  | |  | |  | |  |
| Clinical disease history | In terms of clinical history, we asked whether there were any hypoxic symptoms such as tinnitus, dizziness, limb weakness, shortness of breath | Always =1 | Often =2 | | General =3 | | Occasionally =4 | | Never =5 |
|  | History of intestinal disease, cardiovascular disease, cerebrovascular disease, blood and stomach disease, lung disease, altitude disease, cancer; | The responses were scored from 0 to 5 |  | |  | |  | |  |
|  | Whether they suffered from other common diseases in the past two weeks | The responses were scored from 0 to 5 |  | |  | |  | |  |
|  | The number of hospitalizations in the last two weeks. | The responses were scored from 0 to 5 |  | |  | |  | |  |
